# Effects of Preoperative Statin on Acute Kidney Injury After Off‐Pump Coronary Artery Bypass Grafting

**DOI:** 10.1161/JAHA.118.010892

**Published:** 2019-03-23

**Authors:** Jungchan Park, Jong‐Hwan Lee, Keoung Ah Kim, Seung‐Hwa Lee, Young Tak Lee, Wook Sung Kim, Jeong Jin Min

**Affiliations:** ^1^ Department of Anesthesiology and Pain Medicine Samsung Medical Center Sungkyunkwan University School of Medicine Seoul Korea; ^2^ Department of Thoracic and Cardiovascular Surgery Samsung Medical Center Sungkyunkwan University School of Medicine Seoul Korea; ^3^ Division of Cardiology Department of Medicine Heart Vascular Stroke Institute Samsung Medical Center Sungkyunkwan University School of Medicine Seoul Korea

**Keywords:** acute kidney injury, coronary artery bypass grafting, statin, Cardiovascular Surgery, Complications, Acute Coronary Syndromes

## Abstract

**Background:**

Although many patients with coronary artery disease are using statins before off‐pump coronary artery bypass grafting (OPCAB) following current guidelines, recent studies have raised concerns regarding adverse effects of preoperative statins on postoperative kidney function. We evaluated the effects of preoperative statins on acute kidney injury (AKI) after OPCAB.

**Methods and Results:**

We enrolled 1783 consecutive OPCAB patients in either a statin or nonstatin group based on preoperative use of statins. Propensity scores were used to adjust the differences between the groups. The primary outcome was incidence of postoperative AKI according to Kidney Disease: Improving Global Outcomes criteria. To evaluate the dose‐related renal effects of statins, the statin group was divided into low‐ and moderate‐ or higher dose groups based on preoperative statin dose. The incidence of postoperative AKI was 15.7% and 13.5% in the nonstatin and statin groups, respectively, and preoperative statins did not increase the incidence of postoperative AKI (odds ratio: 0.84; 95% CI, 0.61–1.15; *P*=0.27). In dose‐related analysis, the moderate‐ or higher dose group showed lower incidence of postoperative AKI in comparison with the nonstatin group (odds ratio: 0.61; 95% CI, 0.39–0.95; *P*=0.03). However, no difference was found between low‐dose and nonstatin groups (odds ratio: 1.17; 95% CI, 0.75–1.84; *P*=0.49) or between moderate‐ or higher dose and low‐dose statin groups (odds ratio: 0.84; 95% CI, 0.5–1.41; *P*=0.51) in the incidence of postoperative AKI.

**Conclusions:**

Neither preoperative statin use nor statin dose increased the risk of AKI after OPCAB. Preoperative statin therapy is not harmful in patients receiving OPCAB.

**Clinical Perspective**

**What Is New?**

- This study evaluated whether preoperative statin use affects the occurrence of acute kidney injury after off‐pump coronary artery bypass grafting.
- Neither preoperative statin use nor statin dose increased the risk of acute kidney injury.

**What Are the Clinical Implications?**

- The results of this study show that preoperative statin use is not harmful for patients undergoing off‐pump coronary artery bypass grafting.
- Considering the benefits of statins in cardiovascular protection, preoperative statin therapy according to current guidelines is not harmful for patients receiving off‐pump coronary artery bypass grafting.

## Introduction

Because current guidelines recommend the use of statins for adults with increased risk of cardiovascular disease,^1^ many patients with coronary artery disease take statins before percutaneous coronary intervention or coronary artery bypass grafting (CABG). Statins are known to attenuate perioperative inflammation and oxidative stress, which have been suggested as components of the mechanism for postoperative acute kidney injury (AKI).^2^, ^3^

The potential renoprotective effects of statins in cardiac surgery have been evaluated in numerous studies; however, the results were inconsistent among studies with different settings.^4^, ^5^, ^6^, ^7^, ^8^, ^9^, ^10^, ^11^ In addition, several previous studies have evaluated the dose‐related effects of statins on kidney function after various procedures. In cardiac interventions, it has been suggested that higher doses of statins were more protective against contrast‐induced nephropathy.^12^ In contrast, no association has been observed between statin dosage and postoperative AKI in cardiac surgery.^13^, ^14^ Moreover, recent studies have raised concerns regarding the adverse effects of preoperative statins on the occurrence of AKI after cardiac surgery.^10^, ^15^, ^16^, ^17^

Off‐pump CABG (OPCAB) is a surgical procedure that is not affected by cardiopulmonary bypass; therefore, the effects of statins on AKI after OPCAB would be different from those after percutaneous coronary intervention or conventional CABG. Considering that postoperative AKI is a serious complication that increases mortality^18^, ^19^, ^20^ and morbidity^21^, ^22^ in cardiac surgical patients, the exact effects of preoperative statins on AKI after OPCAB need to be determined. The aim of this study was to evaluate the effects of preoperative statin use and dose on the incidence of AKI after OPCAB.

## Methods

The data will be available to other researchers for the purposes of reproducing our results upon request to the corresponding author.

### Study Population

This study was approved by the institutional review board of Samsung Medical Center (IRB no. 2016‐11‐066‐001) and conducted in accordance with the principles of the Declaration of Helsinki. Because this retrospective study reviewed electronic medical records, individual consent was waived. In total, 1908 adult patients who underwent OPCAB consecutively at our institution from January 2010 to June 2015 were selected, and 125 patients who required intraoperative cardiopulmonary bypass were excluded. Finally, 1783 patients were enrolled as the study population and grouped into either the statin or nonstatin group based on use of preoperative statins. To evaluate the dose‐related renal effects of preoperative statins, the statin group was further divided into low‐dose and moderate‐ or higher dose statin groups based on preoperative statin dose.

The statin doses used for patient grouping were in accordance with the previously reported lipid‐lowering effect of statins.^1^, ^23^ In brief, ≥10 mg of rosuvastatin, 20 mg of atorvastatin, or 40 mg of simvastatin was defined as moderate or high dose (*moderate/high dose*), and all lower statin doses were categorized as *low dose*. All participants were deidentified and analyzed anonymously.

### Data Collection

From January 2017 to July 2017, perioperative medical data were collected using a standardized form and protocol. For all patients scheduled for surgery, it was our institutional policy to investigate all current medications and convert them to in‐hospital prescriptions. After the admission, the departments of surgery and anesthesiology independently investigated current medications. After curating perioperative medications from both departments, nursing records were additionally investigated to minimize missing data. Perioperative medications, laboratory findings, and echocardiographic data were extracted automatically from the electronic medical records with the aid of the hospital's medical information department. After OPCAB, all patients were immediately transferred to the intensive care unit and were closely monitored. Postoperative outcome data were collected though manual review of each case by 2 independent researchers who were blinded to the preoperative statin therapy.

### Study Outcomes and Definitions

The primary outcome was incidence of postoperative AKI, as defined by the Kidney Disease: Improving Global Outcomes criteria using creatinine level.^24^ In brief, occurrence of AKI was defined as either an increase in serum creatinine ≥0.3 mg/dL within 48 hours postoperation or an increase to ≥1.5 times baseline within 7 days. Secondary outcomes were in‐hospital major adverse cardiovascular and cerebral events (MACCE) and newly appearing atrial fibrillation. The durations of intensive care and in‐hospital stay were also compared.

MACCE was defined as a composite of all‐cause death, myocardial infarction (MI), and stroke. MI was defined according to Third Universal Definition of Clinically Relevant MI.^25^ Stroke was defined as a new ischemic or hemorrhagic lesion with a neurological deficit lasting >24 hours. The durations of intensive care and in‐hospital stay were also compared. Chronic kidney disease was defined as serum creatinine >2.0 mg/dL or on dialysis.

The EuroSCORE II was calculated using an online calculator based on the risk model.^26^

### Statistical Analysis

To reduce selection bias and preoperative confounding factors between the statin and nonstatin groups, we performed rigorous adjustment using propensity score matching on all variables associated with preoperative statin use or postoperative outcome. To estimate the propensity scores, the following covariates were included in the model: age; sex; body mass index; smoking; comorbidities (eg, diabetes mellitus, hypertension, stroke, chronic kidney disease, dialysis, and acute MI); preoperative medications (eg, β‐blockers, calcium channel blockers, angiotensin‐converting enzyme inhibitors, angiotensin receptor II blockers, aspirin, and clopidogrel); preoperative left ventricular ejection fraction; hemoglobin, platelet and serum albumin levels; and intraoperative parameters including number of anastomoses, aortic manipulation, operative duration, inotropic requirement at the end of surgery, infused units of red blood cells, and urine output. A propensity score analysis using greedy 1:1 nearest neighbor matching was implemented through the MatchIt package in R (R Foundation for Statistical Computing). After propensity matching, the balance between the 2 groups was evaluated using the standardized difference. An absolute standardized mean difference <10% for the measured covariates was considered an appropriate balance.

In the propensity‐matched population, clinical outcomes were compared between the statin and nonstatin groups. To assess the dose‐related renal effects of preoperative statins, the clinical outcomes of the low‐ and moderate/high‐dose statin groups were compared with those of the nonstatin group. In the subanalysis of the propensity‐matched populations, the low‐dose statin group was matched to the moderate/high‐dose statin group using propensity scores, and clinical outcomes were compared.

For study outcome analysis, we used a logistic regression model with robust variance and reported the odds ratios (ORs) with 95% CIs. To provide power to detect the difference between statin groups, statistical power was calculated based on the number of analyzed patients (1286 patients) and the OR for the primary outcome. In addition, the effect sizes were also computed using Cohen's *h* in the entire and propensity‐matched populations.

For continuous variables, we used the paired *t* test, the Wilcoxon rank sum test, the Wilcoxon signed rank test, or ANOVA to assess the significant differences between groups, and the results are presented as mean±SD. The χ^2^ test or McNemar test was used for categorical data. To assess the independent risk factors for postoperative AKI, univariate and multivariable logistic regression models were constructed. Variables that were clinically relevant or that had *P*<0.1 in univariate analysis were entered into the multivariable logistic regression model. All statistical analyses were performed using SAS software v9.4 (SAS Institute). All tests were 2‐tailed, and *P*<0.05 was considered statistically significant.

## Result

### Patient Characteristics

The baseline characteristics of the entire group and the propensity‐matched populations are shown in Table 1. Of the enrolled 1783 patients, 967 (54.2%) were exposed and 816 (45.8%) were not exposed to preoperative statin therapy. The mean EuroSCORE II values of the patients were 4.0±1.7% in the statin group and 4.1±2.0% in the nonstatin group (*P*=0.16).^26^ After 1:1 individual matching based on propensity score, 643 pairs were generated. The 2 groups contained some mismatched baseline characteristics including smoking history; presence of hypertension, stroke, and acute MI; and preoperative usage of β‐blockers, calcium channel blockers, angiotensin‐converting enzyme inhibitors, angiotensin II receptor blockers, aspirin, and clopidogrel (standardized mean difference >10%;). However, there were no significant differences between the study groups in the propensity score‐matched populations.

**Table 1:** Baseline Characteristics of Entire and Propensity‐Matched Populations

	Entire Population				Propensity‐Matched Population			
	Statin Group (n=967)	Nonstatin Group (n=816)	*P* Value	SMD	Statin Group (n=643)	Nonstatin Group (n=643)	*P* Value	SMD
Male	755 (78.1)	633 (77.6)	0.8	1.2	506 (78.7)	502 (78.1)	0.79*	1.5
Age, y	63.4±9.8	63.3±10.2	0.84	0.7	63.5±9.7	63.5±10.2	0.94*	0.4
BMI, kg/m^2^	24.7±3.0	24.7±2.9	0.82	0.6	24.7±3.1	24.7±2.9	0.94*	−0.4
Previous conditions								
Diabetes mellitus	453 (46.9)	348 (42.7)	0.08	8.4	270 (42.0)	287 (44.6)	0.34*	−5.3
Hypertension	631 (65.3)	494 (60.5)	0.04	9.9	388 (60.3)	398 (61.9)	0.56*	−3.3
Stroke	133 (13.8)	72 (8.8)	0.001	14.3	73 (11.4)	67 (10.4)	0.58*	2.7
Chronic kidney disease	54 (5.6)	34 (4.2)	0.17	6.2	30 (4.7)	33 (5.1)	0.6*	−3.3
Serum creatinine	1.10±1.07	1.25±1.49	0.02		1.14±1.14	1.14±1.17	0.24†	
Dialysis	32 (3.3)	20 (2.5)	0.28	4.8	16 (2.5)	20 (3.1)	0.51*	−3.5
Acute MI	94 (9.7)	105 (12.9)	0.04	−10.6	68 (10.6)	72 (11.2)	0.72*	−2.1
Smoking	293 (30.3)	301 (36.9)	0.003	−14.3	212 (33.0)	221 (34.4)	0.60*	−3
Ejection fraction, %	56.8±12.5	56.1±13.1	0.34	6	56.1±12.9	56.4±12.7	0.71†	−3.1
Medication								
β‐Blocker	408 (42.2)	140 (17.2)	<0.001	50.7	155 (24.1)	140 (21.8)	0.08*	4.7
CCB	272 (28.1)	175 (21.5)	0.001	14.9	152 (23.6)	156 (24.3)	0.79*	−1.4
ACEI	137 (14.2)	57 (7.0)	<0.001	20.6	63 (9.8)	56 (8.7)	0.48*	3.1
ARB	234 (24.2)	124 (15.2)	<0.001	21.3	117 (18.2)	116 (18.0)	0.94*	0.4
Aspirin	876 (90.6)	652 (80.0)	<0.001	36.6	564 (87.7)	565 (87.9)	0.92*	−0.5
Clopidogrel	635 (65.7)	429 (52.6)	<0.001	27.6	397 (61.7)	399 (62.1)	0.89*	−0.7
Blood test								
Hemoglobin, g/dL	13.1±1.8	13.2±1.9	0.07	−8.3	13.2±1.8	13.2±1.9	0.98*	0.1
Platelet, 10^3^/μL	213.1±59.7	214.6±56.9	0.37	−2.4	213.3±61.7	214.4±56.9	0.56†	−1.7
Albumin, g/dL	4.20±0.40	4.14±0.41	0.002	14.4	4.18±0.41	4.17±0.40	0.63†	2.3
Intraoperative parameter								
Anastomosis number	3.9±1.3	4.0±1.4	0.79	−1.9	3.9±1.3	4.0±1.4	0.68†	−3.5
Aortic manipulation	105 (11.0)	91 (11.2)	0.90	−0.6	62 (9.6)	68 (10.6)	0.58	−3.0
Operative duration, h	5.6±1.3	5.7±1.2	0.07	−7.6	5.6±1.3	5.6±1.2	0.58*	−3.1
Inotropic use at end	700 (72.4)	548 (67.2)	0.02	0.12	452 (70.3)	447 (69.5)	0.76*	1.7
Packed RBCs, U	2.2±1.5	2.2±1.7	0.16	2.8	2.2±1.5	2.2±1.7	0.4†	−3.8
Urine output, mL	919±680	900±648	0.82	2.8	915±645	900±652	0.76†	−2.8

Values are n (%) or mean±SD. ACEI indicates angiotensin‐converting enzyme inhibitor; ARB, angiotensin II receptor blocker; BMI, body mass index; CCB, calcium channel blocker; MI, myocardial infarction; RBC, red blood cell; SMD, standardized mean difference.

For continuous variables, Wilcoxon rank sum test, paired t test* (age, BMI, hemoglobin, operative duration), or Wilcoxon signed rank test^†^ was used.

For categorical variables, χ^2^ or McNemar test* (male, diabetes, hypertension, stroke, chronic kidney disease, dialysis, smoking, medications, inotropic use) was used.

Among the 643 patients matched in the statin group, 344 (53.5%) with moderate/high‐dose statins and 299 (46.5%) with low‐dose statins were identified. Detailed data regarding the types and doses of statins are presented in Table S1. The baseline characteristics of the 3 groups according to preoperative statin dose are presented in Table 2.

**Table 2:** Baseline Characteristics of Propensity‐Matched Population According to Statin Dose

	Nonstatin Group (n=643)	Low‐Dose Group (n=299)	Moderate/High‐Dose Group (n=344)	*P* Value	
				Nonstatin vs Low	Nonstatin vs Moderate/High
Male	502 (78.1)	239 (79.9)	267 (77.6)	0.52	0.87
Age, y	63.5±10.2	63.9±9.3	63.2±10.0	0.74	0.55
BMI, kg/m^2^	24.7±2.9	24.5±2.9	24.8±3.3	0.33*	0.64
Previous conditions					
Diabetes mellitus	287 (44.6)	127 (42.5)	143 (41.6)	0.53	0.35
Hypertension	398 (61.9)	192 (64.2)	196 (57.0)	0.49	0.13
Stroke	67 (10.4)	34 (11.4)	39 (11.3)	0.66	0.66
Chronic kidney disease	33 (5.1)	22 (7.4)	8 (2.3)	0.18	0.04
Serum creatinine	1.14±1.17	1.23±1.33	1.06±0.95	0.07	0.92
Dialysis	20 (3.1)	12 (4.0)	4 (1.2)	0.48	0.06
Acute MI	72 (11.2)	27 (9.0)	41 (11.9)	0.31	0.73
Smoking	221 (34.4)	101 (33.8)	111 (32.3)	0.86	0.51
Ejection fraction, %	56.4±12.7	56.2±12.0	56.0±13.7	0.53	0.99
Medication					
β‐Blocker	140 (21.8)	70 (23.4)	85 (24.7)	0.57	0.29
CCB	156 (24.3)	75 (25.1)	77 (22.4)	0.78	0.51
ACEI	56 (8.7)	26 (8.7)	37 (10.8)	0.99	0.29
ARB	116 (18.0)	65 (21.7)	52 (15.1)	0.18	0.24
Aspirin	565 (87.9)	270 (90.3)	294 (85.5)	0.27	0.28
Clopidogrel	399 (62.1)	194 (64.9)	203 (59.0)	0.40	0.35
Blood test					
Hemoglobin, g/dL	13.2±1.9	13.2±1.9	13.1±1.7	0.82	0.57
Platelet, 10^3^/μL	214.4±56.9	212.7±62.1	213.9±61.5	0.28	0.92
Albumin, g/dL	4.17±0.40	4.16±0.43	4.20±0.40	0.67	0.14
Intraoperative parameter					
Anastomosis number	4.0±1.4	4.0±1.3	3.9±1.3	0.62	0.30
Aortic manipulation	68 (10.6)	29 (9.7)	33 (9.6)	0.68	0.63
Operative duration, h	5.6±1.2	5.6±1.3	5.5±1.2	0.83	0.32
Inotropic use at end	447 (69.5)	214 (71.6)	238 (69.2)	0.52	0.91
Packed RBCs, U	2.2±1.7	2.3±1.5	2.1±1.5	0.21	0.43
Urine output, mL	900±652	915±667	915±626	0.93	0.59

Values are n (%) or mean±SD. For categorical variables, McNemar test was used. ACEI indicates angiotensin‐converting enzyme inhibitor; ARB, angiotensin II receptor blocker; BMI, body mass index; CCB, calcium channel blocker; MI, myocardial infarction; RBC, red blood cell.

For continuous variables, Wilcoxon signed rank sum test or paired *t* test* was used.

### Preoperative Statins and Postoperative AKI

The overall incidence of postoperative AKI was 14.6% (188/1286). The incidence of postoperative AKI was 15.7% and 13.5% in the nonstatin and statin groups, respectively, and preoperative statin use did not increase the incidence of postoperative AKI (OR: 0.84; 95% CI, 0.61–1.15; *P*=0.27; Table 3). Based on this result and the number of analyzed patients, the statistical power was calculated as 0.2027, and the effect sizes using Cohen's *h* were 0.094 in the entire population and 0.061 in the propensity‐matched population, suggesting that there was no significant effect of preoperative statin use on the incidence of postoperative AKI.^27^

**Table 3:** Clinical Outcomes of Statin Group Versus Nonstatin Groups in Propensity‐Matched Population

	Nonstatin (n=643)	Statin (n=643)	OR (95% CI)	*P* Value
AKI				
Overall	101 (15.7)	87 (13.5)	0.84 (0.61–1.15)	0.27
Stage 1	87 (13.5)	82 (12.8)	0.93 (0.67–1.29)	0.68
Stage 2	6 (0.9)	2 (0.3)	0.33 (0.07–1.65)	0.18
Stage 3	8 (1.2)	3 (0.5)	0.38 (0.10–1.41)	0.15
MACCE				
Overall	13 (2.0)	10 (1.6)	0.77 (0.34–1.75)	0.53
Death	1 (0.2)	4 (0.6)	4.00 (0.45–35.8)	0.22
MI	7 (1.1)	1 (0.3)	0.14 (0.02–1.16)	0.07
Stroke	5 (0.8)	5 (0.8)	1.00 (0.29–3.45)	>0.99
Atrial fibrillation	104 (16.2)	95 (148)	0.90 (0.67–1.22)	0.49
ICU care duration, h	48.6±68.9	45.8±55.4	…	0.43^a^
In‐hospital duration, d	8.4±10.7	8.9±12.9	…	0.47^a^

Values are n (%) or mean±SD. AKI indicates acute kidney injury; ICU, intensive care unit; MACCE, major adverse cardiovascular and cerebral events; MI, myocardial infarction; OR, odds ratio.

a Wilcoxon signed rank test.

In further analyses evaluating the dose‐related renal effects of preoperative statins, the moderate/high‐dose group showed lower incidence of postoperative AKI compared with the nonstatin group (OR: 0.61; 95% CI, 0.39–0.95; *P*=0.03). However, no differences were found between the low‐dose and nonstatin groups (OR: 1.17; 95% CI, 0.75–1.84; *P*=0.49; Table 4). The effects of preoperative statins on each stage of postoperative AKI showed no significant association between preoperative statin dose and risk of any specific stage of postoperative AKI (Table 4).

**Table 4:** Dose‐Related Effects of Preoperative Statins on Postoperative Clinical Outcomes

	Statin Dose	n/N (%)	OR (95% CI)	*P* Value
AKI				
Overall	Nonstatin	101/643 (15.7)	1	
	Low dose	47/299 (15.7)	1.17 (0.75–1.84)	0.49
	Moderate/high dose	40/344 (11.6)	0.61 (0.39–0.95)	0.03
Stage 1	Nonstatin	87/643 (13.5)	1	
	Low dose	43/299 (14.4)	1.27 (0.79–2.04)	0.71
	Moderate/high dose	39/344 (11.3)	0.71 (0.45–1.12)	0.14
Stage 2	Nonstatin	7/643 (1.1)	1	
	Low dose	2/299 (0.6)	1.00 (0.07–13.80)	0.99
	Moderate/high dose	0/344 (0)	0.19 (0.00–1.12)	0.99
Stage 3	Nonstatin	8/643 (1.2)	1	
	Low dose	2/299 (0.7)	0.50 (0.09–2.73)	0.42
	Moderate/high dose	1/344 (0.3)	0.25 (0.03–2.24)	0.22
MACCE				
Overall	Nonstatin	13/643 (2.0)	1	
	Low dose	4/299 (1.3)	1.00 (0.25–4.00)	0.99
	Moderate/high dose	6/344 (1.7)	0.67 (0.24–1.87)	0.44
Death	Nonstatin	1/643 (0.2)	1	
	Low dose	2/299 (0.7)	2.41 (0.29 to >99.99)	0.99
	Moderate/high dose	2/344 (0.6)	2.00 (0.10–117.99)	0.57
MI	Nonstatin	7/643 (1.1)	1	
	Low dose	0/299 (0)	0.26 (<0.01–1.71)	0.99
	Moderate/high dose	1/344 (0.3)	0.25 (0.01–2.53)	0.22
Stroke	Nonstatin	5/643 (0.8)	1	
	Low dose	2/299 (0.7)	2.00 (0.18–22.06)	0.57
	Moderate/high dose	3/344 (0.9)	0.75 (0.17–3.35)	0.71
Atrial fibrillation	Nonstatin	104/643 (16.2)	1	
	Low dose	45/299 (15.1)	0.83 (0.53–1.31)	0.43
	Moderate/high dose	50/344 (14.5)	0.96 (0.64–1.44)	0.83

AKI indicates acute kidney injury; MACCE, major adverse cardiovascular and cerebral events; MI, myocardial infarction; OR, odds ratio.

### Predictors of Postoperative AKI

In the multivariable logistic regression analysis to identify predictors for postoperative AKI in the entire population, preoperative use of moderate/high‐dose statins was not associated with risk of postoperative AKI after adjusting for variables with *P*<0.1 in the univariate analysis (OR: 0.78; 95% CI, 0.55–1.12; *P*=0.18). Patient age, presence of preoperative hypertension or chronic kidney disease, low left ventricular ejection fraction, nonuse of β‐blockers, low preoperative hemoglobin level, prolonged operative duration, and inotropic requirement at the end of surgery independently increased the risk of postoperative AKI (Table 5).

**Table 5:** Predictors of Postoperative AKI in the Entire Population

	Unadjusted Analysis		Adjusted Analysis	
	OR (95% CI)	*P* Value	OR (95% CI)	*P* Value
Moderate/high‐dose statin	0.67 (0.49–0.92)	0.01	0.78 (0.55–1.12)	0.18
Male	0.96 (0.70–1.32)	0.81		
Age	1.04 (1.02–1.05)	<0.001	1.03 (1.01–1.05)	0.002
BMI	0.95 (0.91–1.00)	0.05	1.01 (0.96–1.07)	0.70
Previous conditions				
Diabetes mellitus	1.80 (1.37–2.34)	<0.001	1.13 (0.83–1.55)	0.43
Hypertension	2.14 (1.57–2.93)	<0.001	2.02 (1.40–2.92)	<0.001
Stroke	1.20 (0.80–1.78)	0.38		
Chronic kidney disease	10.61 (6.79–16.58)	<0.001	4.72 (2.75–8.10)	<0.001
Acute MI	1.88 (1.31–2.72)	0.001	1.14 (0.74–1.76)	0.56
Smoking	0.98 (0.74–1.30)	0.9		
Ejection fraction	0.96 (0.95–0.97)	<0.001	0.97 (0.96–0.98)	<0.001
Medication				
β‐Blocker	0.63 (0.46–0.86)	0.004	0.48 (0.33–0.69)	<0.001
CCB	1.36 (1.02–1.83)	0.04	1.23 (0.87–1.74)	0.25
ACEI	0.76 (0.78–1.22)	0.26	…	…
ARB	1.20 (0.87–1.65)	0.27	…	…
Aspirin	0.92 (0.63–1.34)	0.92	…	…
Clopidogrel	0.96 (0.74–1.27)	0.79	…	…
Blood test				
Hemoglobin, g/dL	0.69 (0.64–0.74)	<0.001	0.77 (0.70–0.85)	<0.001
Albumin, g/dL	0.38 (0.28–0.52)	<0.001	0.85 (0.61–1.19)	0.35
Intraoperative parameter				
Anastomosis number	1.10 (0.99–1.22)	0.07	0.99 (0.88–1.11)	0.88
Aortic manipulation	1.79 (1.24–2.60)	0.002	1.26 (0.83–1.93)	0.28
Operative duration, h	1.31 (1.18–1.44)	<0.001	1.26 (1.12–1.43)	<0.001
Inotropic use at end	1.83 (1.32–2.54)	<0.001	1.54 (1.07–2.23)	0.02
Packed RBCs, U	1.18 (1.09–1.28)	<0.001	0.92 (0.83–1.02)	0.11

ACEI indicates angiotensin‐converting enzyme inhibitor; AKI, acute kidney injury; ARB, angiotensin II receptor blocker; BMI, body mass index; CCB, calcium channel blocker; MI, myocardial infarction; OR, odds ratio; RBC, red blood cell.

### Other Postoperative Outcomes

The incidence of postoperative in‐hospital MACCE and newly appearing atrial fibrillation in each statin group is presented in Tables 3 and 4. Compared with nonuse of statins, preoperative statin therapy did not show a significant association with risk of in‐hospital MACCE (or any component thereof) or new‐onset atrial fibrillation regardless of statin dose. The durations (in days) of postoperative intensive care or hospital stay in the nonstatin, low‐dose, and moderate/high‐dose groups were all comparable.

### Subanalysis of Statin Group: Low‐Dose Versus Moderate/High‐Dose

We performed additional propensity score matching to balance the low‐ and moderate/high‐dose groups (Table S2). After 1:1 individual matching according to propensity score, 255 pairs were generated. In the propensity‐matched analysis of the statin groups, there was no difference in the incidence of postoperative AKI between the moderate/high‐ and low‐dose groups (OR: 0.84; 95% CI, 0.5–1.41; *P*=0.51). Other postoperative clinical outcomes, including in‐hospital MACCE and components thereof and newly appearing atrial fibrillation, also did not differ by dose of preoperative statins (Table S3). The duration of postoperative intensive care or hospital stay was also comparable for the low‐ and moderate/high‐dose groups.

## Discussion

In this study, we evaluated the renal effects of preoperative statin therapy and its different doses in patients undergoing OPCAB and found that use of preoperative statins did not increase the incidence of postoperative AKI regardless of dosage. In addition, the incidence of postoperative in‐hospital MACCE and newly appearing atrial fibrillation did not differ based on preoperative statin therapy.

The current guidelines recommend the use of statins, frequently at higher than moderate doses, in adults with increased risk of cardiovascular disease.^1^, ^28^, ^29^, ^30^ Independent of their lipid‐lowering effects, statins exhibit numerous protective effects on the cardiovascular system including improved endothelial function, enhanced stability of atherosclerotic plaques, and decreased oxidative stress and inflammation.^31^, ^32^ When following the guidelines, most patients scheduled for OPCAB are likely to have used statins preoperatively.

Because the main mechanisms of AKI after cardiac surgery include perioperative inflammatory response and oxidative stress,^2^, ^3^ the anti‐inflammatory, antioxidant, and endothelial stabilizing pleiotropic effects of preoperative statins were expected to have a potentially renoprotective role, and those effects have been vigorously investigated in previous clinical studies.^4^, ^5^, ^6^, ^7^, ^8^, ^9^, ^10^, ^11^ However, the results were inconsistent among the studies, and a clear protective effect of preoperative statins on postoperative renal function was not demonstrated in recent randomized trials and meta‐analyses.^11^ These inconsistent results may be partly due to the heterogeneous patient populations among cardiac surgeries, selection bias for preoperative statin use, or varying complexity of the surgical procedures.

Moreover, there have been recent reports regarding unexpected renoadverse effects of statins in surgical or critically ill patients.^10^, ^15^, ^16^, ^17^, ^33^ Suggested undesirable effects of statins on kidney function include increased release of myoglobin from statin‐induced muscle necrosis in patients with kidney disease; insidious induction of tubulointerstitial nephritis; and statin‐induced depletion of coenzyme Q10, which is essential for mitochondrial function and energy production.^34^, ^35^, ^36^, ^37^, ^38^ However, the exact mechanism of statin‐related renoadverse effects seems to be multifactorial and is still unclear. Consequently, concerns remain for patients on moderate or high statin doses, and these concerns may have been reflected in the underuse of statins in previous statin‐related studies on cardiac surgeries.^10^, ^39^ Participants in our study also showed a relatively lower rate of statin use than expected.

In our study, preoperative statin use and dose did not increase the risk of postoperative AKI after OPCAB. A number of new methods were adopted in this study to evaluate the effects of preoperative statins on postoperative renal function. First, unlike previous studies that considered numerous types of cardiac surgeries, our study population was limited to OPCAB patients. In prior studies that compared clinical outcomes of OPCAB versus conventional CABG, the use of the off‐pump technique reduced the incidence of mild renal injury.^40^, ^41^ However, patients at more advanced stages of postoperative renal injury requiring new renal replacement therapy demonstrated no difference regarding the 2 techniques,^42^, ^43^ and the incidence of AKI after OPCAB has been reported to be in the range of 8% to 38%.^40^, ^41^, ^42^, ^44^, ^45^ In the present study, our population included only OPCAB patients with propensity score matching; therefore, the degree of surgical complexity and the patient characteristics were relatively homogeneous, and pump‐related adverse renal effects could be excluded. Second, in addition to comparing statin groups with different doses with the nonstatin group, we performed another comparison of the different doses of statins with additional propensity matching because high‐dose statins were previously reported to have more renoadverse effects than low‐dose statins.^17^ In our study, no difference was found in the incidence of postoperative AKI between the moderate/high‐ and low‐dose groups. Therefore, we could not determine a dose‐related renal effect of statin in OPCAB, although the moderate/high‐dose group showed lower incidence of postoperative AKI than the nonstatin group.

In terms of postoperative outcomes other than AKI, preoperative statin dosage did not affect the incidence of in‐hospital MACCE or new‐onset atrial fibrillation. Owing to the conflicting results of previous studies, the overall effects of statins on postoperative clinical outcome are still under debate.^46^ Unlike observational studies showing the protective effects of statins, studies on isolated CABG and meta‐analyses of randomized trials have failed to demonstrate that preoperative statins improve postoperative clinical outcomes.^16^ In this study, we could not address the effects of preoperative statins on long‐term clinical outcomes because almost all patients started statin therapy within a few days after OPCAB.

This study has strength in terms of clinical implications. Although many randomized trials have focused on the effects of pretreatment with a single type of statin on various cardiac surgeries, we included only a homogeneous OPCAB procedure and various types of statins. This design may not be feasible in randomized studies; however, it is easier to translate into real clinical practice. Although, the current guidelines suggest continuous use of statins in patients undergoing CABG,^47^, ^48^ the prescription rate of statins still tends to be low.^39^, ^49^ The results of our study suggest that statin therapy in compliance with existing guidelines would be reasonable and safe for OPCAB in terms of postoperative renal function.

A limitation of this study is the nature of retrospective observational studies. Even with adjustment using propensity score matching, hidden bias and unmeasured confounding factors could have influenced the results. Second, the type and duration of preoperative statin were not independently evaluated, and only the dose of preoperative statin, classified according to lipid‐lowering effects, was analyzed. Our results were derived only from OPCAB; therefore, different results might be found in conventional CABG. Finally, we could not perform independent analysis for the high‐dose statin group because a limited number of patients were on preoperative high‐dose statin in our study population. Further study independently analyzing the effects of high‐dose preoperative statins in the OPCAB population is required. Despite these limitations, this study is the first to evaluate the renal effects of different doses of preoperative statins in patients undergoing OPCAB.

## Conclusions

Preoperative statin use and dose did not increase the incidence of postoperative AKI after OPCAB. Considering the benefits of statins for cardiovascular protection, preoperative statin therapy according to the current guidelines is not harmful in patients receiving OPCAB.

## Disclosures

None.

## Supporting information


**Table S1.** Types and Doses of Statin
**Table S2.** Baseline Characteristics of Moderate/High‐Dose Versus Low‐Dose Statin Groups
**Table S3.** Clinical Outcomes of Moderate/High‐Dose Versus Low‐Dose Statin Groups in Matched Analysis
