# Supplementary material for: Effects of Preoperative Statin on Acute Kidney Injury After Off‐Pump Coronary Artery Bypass Grafting
Source: J Am Heart Assoc. 2019 Mar 23;8(7):e010892. doi: 10.1161/JAHA.118.010892 (PMC6509717; doi:10.1161/JAHA.118.010892)
Supplement: Supplementary file 1 — Table S1. Types and Doses of Statin Table S2. Baseline Characteristics of Moderate/High‐Dose Versus Low‐Dose Statin Groups Table S3. Clinical Outcomes of Moderate/High‐Dose Versus Low‐Dose Statin Groups in Matched Analysis [file JAH3-8-e010892-s001.pdf]

SUPPLEMENTAL MATERIAL

Table S1. Types and Doses of Statin.

Statin type, dose	N (%)
Low-dose (N = 344)	
Rosuvastatin, 5 mg	13 (3.8)
Atorvastatin, 10 mg	203 (59.0)
Simvastatin, 10 mg	14 (4.1)
Simvastatin, 20 mg	69 (20.1)
Pravastatin, 10 mg	21 (6.1)
Pravastatin, 20 mg	24 (7.0))
Moderate-or High-dose (N = 299)	
Rosuvastatin, 10 mg	88 (29.4)
Rosuvastatin, 20 mg	33 (11.0)
Atorvastatin, 20 mg	101 (33.8)
Atorvastatin, 40 mg	50 (16.7)
Atorvastatin, 80 mg	18 (6.0)
Simvastatin, 40 mg	9 (3.0)

Table S2. Baseline Characteristics of Moderate-or High-Dose Statin Group vs. Low-Dose Statin Group.

	Entire population			Propensity matched population		
	≥ Moderate-dose (N = 344)	Low-dose (N = 299)	SMD	≥ Moderate-dose (N = 255)	Low-dose (N = 255)	SMD
Male	267 (77.6)	239 (79.9)	-5.5	205 (80.4)	206 (80.8)	-0.9
Age	63.2 (±10.0)	63.9 (±9.3)	-7.6	63.2 (±10.0)	63.5 (±9.7)	-2.9
BMI, kg/m^2	24.8 (±3.3)	24.5 (±2.9)	10.7	24.8 (±3.2)	24.6 (±2.8)	5.7
Previous conditions						
Diabetes	143 (41.6)	127 (42.5)	-1.8	107 (42.0)	104 (40.8)	2.4
Hypertension	196 (57.0)	192 (64.2)	-14.6	152 (59.6)	153 (60.0)	-0.8
Stroke	39 (11.3)	34 (11.47)	-0.1	28 (11.0)	27 (10.6)	1.2
Chronic kidney disease	8 (2.3)	22 (7.4)	-33.3	8 (3.1)	10 (3.9)	-5.2
Dialysis	4 (1.2)	12 (4.0)	-26.6	4 (1.6)	5 (2.0)	-3.7
Acute MI	41 (11.9)	27 (9.0)	8.9	24 (9.4)	25 (9.8)	-1.2
Smoking	111 (32.3)	101 (33.8)	-3.2	90 (35.3)	89 (34.9)	0.8
Ejection fraction, %	56.0 (±13.7)	56.2 (±12.0)	-1.3	55.6 (±13.7)	56.2 (±11.5)	-4.7
Medication						
Beta blocker	85 (24.7)	70 (23.4)	3.0	60 (23.5)	61 (23.9)	-0.9
CCB	77 (22.4)	75 (25.1)	-6.5	69 (27.1)	61 (23.9)	7.5
ACEi	37 (10.8)	26 (8.7)	6.6	28 (11.0)	23 (9.0)	6.3
ARB	52 (15.1)	65 (21.7)	-18.5	48 (18.8)	45 (17.7)	3.3
Aspirin	294 (85.5)	270 (90.3)	-13.7	222 (87.1)	226 (88.6)	-4.4
Clopidogrel	203 (59.0)	194 (64.9)	-11.9	159 (62.4)	162 (63.5)	-2.4
Blood test						
Hemoglobin	13.1 (±1.7)	13.2 (±1.9)	-1.0	13.3 (±1.7)	13.2 (±1.8)	6.6
Platelet	213.9 (±61.5)	212.7 (±62.1)	2.0	213.5 (±52.9)	210.5 (±57.4)	4.9
Albumin	4.20 (±0.40)	4.16 (±0.43)	12.1	4.19 (±0.41)	4.18 (±0.40)	2.8
Intraoperative parameter						
Anastomosis number	3.9 (±1.3)	4.0 (±1.3)	-9.5	4.0 (±1.3)	3.9 (±1.3)	0.3

Aortic manipulation	33 (9.6)	29 (9.7)	-0.4	23 (9.0)	24 (9.4)	-1.3
Operative duration, hours	5.5 (±1.2)	5.6 (±1.3)	-7.5	5.6 (±1.2)	5.6 (±1.3)	-0.7
Inotropic use at end	238 (69.2)	214 (71.6)	-5.2	175 (68.6)	181 (71.0)	-5.1
packed RBC, unit	2.1 (±1.5)	2.3 (±1.5)	-13.6	2.1 (±1.5)	2.2 (±1.5)	-8.6
Urine output, ml	915 (±626)	915 (±667)	0.04	909 (±632)	925 (±653)	-2.7

Values are n (%) or mean (±SD)

BMI = Body mass index; MI = Myocardial infarction; CCB = Calcium channel blocker; ACEi = Angiotensin-converting enzyme inhibitor; ARB = Angiotensin II receptor blocker, RBC = red blood cell

Table S3. Clinical Outcomes of Moderate-or High-Dose Statin Group vs. Low-Dose Statin Group in Matched Analysis.

	≥ Moderate-dose (n = 255)	Low-dose (n = 255)	Odds Ratio (95% CI)	*p* Value
AKI				
Any	29 (11.4)	34 (13.3)	0.84 (0.50-1.41)	0.51
Stage 1	29 (11.4)	32 (12.6)	0.90 (0.53-1.52)	0.69
Stage 2	0	1 (0.4)		
Stage 3	0	1 (0.4)		
MACCEs	3 (1.2)	3 (1.2)	1.00 (0.20-4.96)	>0.99
Death	2 (0.8)	2 (0.8)	1.00 (0.14-7.10)	>0.99
MI	0	0		
Stroke	1 (0.4)	1 (0.4)	1.00 (0.06-16.0)	>0.99
New-onset atrial fibrillation	35 (13.7)	38 (14.9)	0.90 (0.54-1.51)	0.69
Intensive care duration, hours	46.4 (± 68.1)	43.0 (± 49.2)		0.52
In-hospital duration, days	9.4 (± 14.1)	8.3 (± 14.0)		0.35

Values are n (%) or mean (±SD)

AKI = acute kidney injury; MACCEs = major adverse cardiovascular and cerebral events; MI = myocardial infarction
